# Evaluation of an ASFV RNA Helicase Gene A859L for Virus Replication and Swine Virulence

**DOI:** 10.3390/v14010010

**Published:** 2021-12-21

**Authors:** Elizabeth Ramirez-Medina, Elizabeth A. Vuono, Sarah Pruitt, Ayushi Rai, Nallely Espinoza, Lauro Velazquez-Salinas, Douglas P. Gladue, Manuel V. Borca

**Affiliations:** 1 Plum Island Animal Disease Center, USDA, Agricultural Research Service, Orient, NY 11944, USA; Elizabeth.Ramirez@usda.gov (E.R.-M.); Elizabeth.Vuono@usda.gov (E.A.V.); Sarah.Pruitt@usda.gov (S.P.); ayushi.rai@usda.gov (A.R.); Nallely.Espinoza@usda.gov (N.E.); lauro.velazquez@usda.gov (L.V.-S.); 2 Department of Pathobiology and Population Medicine, Mississippi State University, Starkville, MS 39762, USA; 3 Oak Ridge Institute for Science and Education (ORISE), Oak Ridge, TN 37830, USA

**Keywords:** ASFV, ASF, African swine fever virus, A859L, helicase

## Abstract

African swine fever virus (ASFV) is producing a devastating pandemic that, since 2007, has spread to a contiguous geographical area from central Europe to Asia. In July 2021, ASFV was detected in the Dominican Republic, the first report of the disease in the Americas in more than 40 years. ASFV is a large, highly complex virus harboring a large dsDNA genome that encodes for more than 150 genes. The majority of these genes have not been functionally characterized. Bioinformatics analysis predicts that ASFV gene A859L encodes for an RNA helicase, although its function has not yet been experimentally assessed. Here, we evaluated the role of the A859L gene during virus replication in cell cultures and during infection in swine. For that purpose, a recombinant virus (ASFV-G-∆A859L) harboring a deletion of the A859L gene was developed using the highly virulent ASFV Georgia (ASFV-G) isolate as a template. Recombinant ASFV-G-∆A859L replicates in swine macrophage cultures as efficiently as the parental virus ASFV-G, demonstrating that the A859L gene is non-essential for ASFV replication. Experimental infection of domestic pigs demonstrated that ASFV-G-∆A859L replicates as efficiently and induces a clinical disease indistinguishable from that caused by the parental ASFV-G. These studies conclude that the predicted RNA helicase gene A859L is not involved in the processes of virus replication or disease production in swine.

## 1. Introduction

African swine fever (ASF) is currently affecting the swine production industry in a large geographical area stretching across Europe and into East and Southeast Asia. In July 2021, the Dominican Republic reported its first outbreak of ASF, which had been absent from North America for 40 years. As a result, global swine industries are threatened by significant economic losses and human populations face food insecurity [1]. Disease control is currently restricted to culling susceptible animals and implementing strict biosecurity measures to prevent disease spread since no commercial vaccines are available. 

The etiological agent, African swine fever virus (ASFV), is a large, structurally complex virus with a large (more than 180 kb pairs) double-stranded DNA genome [2]. ASFV encodes for more than 150 genes, of which most remain uncharacterized [1,2]. Targeted deletion of genes from the genome of ASFV has been a powerful tool to study gene function. The resulting recombinant viruses allow a direct assessment of virus function, particularly in the processes of virus replication and virulence. For instance, the development of ASFV experimental vaccines has focused on the deletion of specific virus genes involved in disease production. The resulting attenuated viruses have been shown effective in preventing disease during challenges with parental virulent strains [3,4,5,6,7]. The development of these vaccines depends on the identification, characterization and genetic manipulation of virus genes involved in virus virulence. Therefore, understanding the function of virus genes in the processes of replication and virulence is critical for the development of experimental vaccines and novel countermeasures [3,4,5,6,7,8].

A small number of ASFV genes have been successfully deleted from the ASFV genome (e.g., TK, NL, CD2, MGF360-16R and 1L, MGF110-1L, L83L, C962R, X69R, I8L) [9,10,11,12,13,14,15,16,17,18], while another small number of genes have been shown essential for virus replication (e.g., EP152R, p30, p54, p72) [19,20,21,22]. These studies demonstrate that deleting specific genes by genetic manipulation of the virus genome is a powerful approach to study the function of a particular gene or group of genes during infection.

Six ASFV genes with potential RNA helicase activity have been identified using functional genomics: QP509L, Q706L, D1133L, B962L, F105L and A859L [23]. The genes QP509L and Q706L have been experimentally assessed using siRNA, demonstrating that siRNA blocking of these genes results in >90% reduction in virus replication in cell cultures [24]. However, it recently has been shown that QP509L is not essential for virus replication [25]. The function of the A859L gene as a predicted RNA helicase has not been experimentally evaluated [24]. This report investigated the importance of the A859L gene during ASFV replication in swine macrophage cultures and during experimental infection in domestic pigs. 

## 2. Materials and Methods

### 2.1. Viruses and Cells

Primary cultures of blood-derived swine macrophages were performed as previously described [26]. Ficoll-Paque (Pharmacia, Piscataway, NJ, USA) density gradients were used to purify blood mononuclear cells, and the obtained adherent cells were seeded into Primaria T25, 6- or 96-well dishes at a density of 5 × 10^6^ cells per ml. ASFV Georgia (ASFV-G) was a field isolate kindly provided by Dr. Nino Vepkhvadze from the Laboratory of the Ministry of Agriculture (LMA) in Tbilisi, Republic of Georgia [27]. Growth curves between ASFV-G-∆A859L and parental ASFV-G were performed in primary swine macrophage cell cultures in 24-well plates at an MOI of 0.01 HAD_50_ (hemadsorbing doses, as determined in primary swine macrophage cell cultures). After adsorption for 1 h at 37 °C under 5% CO_2_, the inoculum was removed, cells rinsed with PBS twice, and further incubated with macrophage media for 2, 24, 48, 72 and 96 h at 37 °C under 5% CO_2_. At described times post-infection, the cells were frozen at ≤−70 °C and thawed, and the lysates were titrated by HAD_50_/_mL_ in primary swine macrophage cell cultures in 96-well plates. All samples were run simultaneously to avoid inter-assay variability. The presence of the virus was assessed by hemadsorption (HA), and virus titers were calculated as previously described [28].

### 2.2. Detection of A859L Transcription 

As previously described [29], we used a real-time PCR assay (qPCR) to evaluate the transcriptional profile of the A859L gene during the infection of ASFV-G in cultures of porcine macrophages, using the early CP204L (p30) and late B646L (p72) expressed genes of ASFV as reference genes. Briefly, cell cultures of porcine macrophages were infected with a stock of ASFV-G using an MOI of 1. RNA extractions using an RNeasy Kit (QIAGEN, Hilden, Germany) were conducted at 4, 6, 8, and 24 h post-infection. All extractions were treated with 2 units of DNase I (BioLabs, San Diego, CA, USA) then purified using the Monarch^®^ RNA Cleanup Kit (New England BioLabs, Inc., Ipswich, MA, USA). One μg of RNA was used to produce cDNA using qScript cDNA SuperMix (Quanta bio, Beverly, MA, USA) that was used for the qPCR. 

Primers and probes for the detection of the A859L gene were designed using the ASFV Georgia 2007/1 strain (GenBank Accession #NC_044959.2). Primer forward: 5′-GTGTGATCTCCCGCCTATG-3′, reverse: 5′-CTTCCACAGGAGTTATCACCAG-3′ and probe: 5′-FAM/AGCCATCTTTGCCCTCTGATCCG /MGBNFQ-3′. Primers and probes for the detection of p72 gene: forward 5′-CTTCGGCGAGCGCTTTATCAC-3′, reverse: 5′-GGAAATTCATTCACCAAATCCTT-3′ and probe: 5′-FAM-CGATGCAAGCTTTAT -MGB NFQ-3′. Primers and probes for the detection of CP204L (p30) gene: forward 5′-GACGGAATCCTCAGCATCTTC-3′, reverse: 5′-CAGCTTGGAGTCTTTAGGTACC-3′ and probe: 5′-FAM-TGTTTGAGCAAGAGCCCTCATCGG-MGB NFQ-3′. Primers and probes for the detection of the β-actin gene: forward 5′-GACCTGACCGACTACCTCATG-3′, reverse: 5′-TCTCCTTGATGTCCCGCAC-3′ and probe: 5′-FAM-CTACAGCTTCACCACCACGGC-MGB NFQ-3′. All qPCRs were conducted using the TaqMan Universal PCR Master Mix (Applied Biosystems, Waltham, MA, USA) using the following amplification conditions: One step at 55 °C for 2 min, followed by one denaturation step at 95 °C for 10 min, then 40 cycles of denaturation at 95 °C for 15 s and annealing/extension at 65 °C for 1 min.

### 2.3. Construction of the ASFV A859L Deletion Mutant 

ASFV lacking the A859L gene (ASFV-G-∆A859L) was developed by homologous recombination between the genome of the parental ASFV-G and a recombination transfer vector as described elsewhere [3]. The recombinant transfer vector (p72mCherryΔA859L) harbors flanking genomic regions of the A859L gene: the left arm is located between genomic positions 51,317–52,317, and the right arm is located between genomic positions 54,758–55,758 and harbors a reporter gene cassette containing the mCherry fluorescent protein (mCherry) gene under the control of the ASFV p72 late gene promoter [30]. The recombinant transfer vector was obtained by DNA synthesis (Epoch Life Sciences, Sugar Land, TX, USA). As designed, this construction created a 2439-nucleotide deletion between nucleotide positions 52,318–54,757, partially deleting the A859L ORF sequence, leaving only the last 138 nucleotides of A859L to not disturb the neighboring gene promoter for A238L. Recombinant mutant ASFV-G-∆A859L was purified to homogeneity by successive rounds of limiting dilution purification based on mCherry activity detection. and was full-length sequenced using next-generation sequencing (NGS).

### 2.4. Next-Generation Sequencing of ASFV

ASFV DNA was extracted from infected cell cultures showing 90–100% CPE, using the nuclear extraction kit (Active Motif cat# 40010), the nucleus and cytoplasmic fractions were separated, the cytoplasmic fraction was used to isolate the viral DNA, following the manufacturer’s protocol. In brief, ASFV infected cells were collected and incubated in the hypotonic buffer for 15 min on ice or until the cell membrane dissolved. The nucleus fraction is separated by centrifugation. The cytoplasmic fraction is collected, and DNA is extracted by adding 10% 3M Na0Ac by volume to the sample (Sigma-Aldrich 71196, St. Louis, MO, USA) and an equal volume of phenol:chloroform:isoamyl alcohol (25:24:1) with a Ph of 6.5–6.9 (Sigma-Aldrich P3803-100ML), then centrifuged for max speed in a tabletop centrifuge. The aqueous layer is then ethanol-precipitated using 2 volumes of 100% ethanol, washed with the same volume of 70% ethanol and dried the resulting DNA pellet is then reconstituted in sterile water. We then used this DNA library for NGS sequencing using Nextera XT kit in the NextSeq (Illumnia, San Diego, CA, USA) following the manufactures protocol. Sequence analysis was performed using CLC Genomics Workbench software (CLCBio, Waltham, MA, USA).

### 2.5. Animal Experiments

The virulence of ASFV-G-∆A859L was evaluated using 35–40 kg commercial breed swine. A group of five pigs was intramuscularly (IM) inoculated with 10^2^ HAD_50_ of ASFV-G-∆A859L and compared with a group of five pigs inoculated with 10^2^ HAD_50_ of ASFV-G. Clinical signs (anorexia, depression, fever, purple skin discoloration, staggering gait, diarrhea and cough) and changes in body temperature were recorded daily throughout the experiment. Blood samples were obtained at 0, 4 and 7 days post-inoculation (pi). Animal experiments were performed under biosafety level 3 conditions in the animal facilities at Plum Island Animal Disease Center, following a strict protocol approved by the Institutional Animal Care and Use Committee (225.01-16-R_090716).

## 3. Results and Discussion

### 3.1. A859L Gene Is Conserved across Different ASFV Isolates

The A859L gene encodes for a virus protein predicted, through functional genomics, to be an RNA helicase [31]. Besides this information, there is no data indicating the transcriptional activity and the function of this virus gene. Proteomic analysis conducted in the A859L protein of the ASFV strain Georgia using the software PFAM version 34.0 (https://pfam.xfam.org/ (accessed on: 22 November 2021) showed the existence of a T5orf172 domain (residues 5 to 78), a type III restriction enzyme subunit (residues 166–332) that contains a DEAD/DEAH box helicase domain (residues 170–332), and followed by a helicase conserved C-terminal domain (residues 399–499). Blast analysis of these domains showed an identity around 50 to 30% with the helicase-like protein of different bacteria and parasite species. 

Interestingly, blast analysis between A859L protein and other predicted RNA helicases in ASFV, such as QP509L and Q706L, indicates that there is a low percentage of identity (28–26% and 30.16%) in the type III restriction enzyme subunit with these proteins. Furthermore, an identity of 32.61% was depicted just between A859L and QP509L proteins in the DEAD/DEAH box helicase domain. No other significant percentages of identity were found in other functional domains, indicating the potential functional differences between A859L and the other two predicted ASFV RNA helicases.

To assess the diversity of the A859L protein across multiple ASFV isolates available through the NCBI GenBank database, we conducted a pairwise comparison analysis. The average A859L protein is 859 residues in length. The % identity at the nucleotide and amino acid levels across multiple ASFV isolates was 90.03–99.96% and 85.56–99.88%, respectively. Overall, 146 variable sites were found in the protein alignment, reflecting the multiple missense mutations accumulated during the evolution of the A859L protein (Supplemental Figure S1). Interestingly, different from most proteins of ASFV belonging to the Eurasia lineage, A859L protein was not 100% conserved among isolates of this lineage. We found two variable sites at positions A149T and A427E associated with changes in the ASFV isolates Estonia 2014 and Tanzania/Rukwa 2017, respectively. 

To gain more insight into the potential relevance of this variability, we conducted the evolutionary test Mixed Effects Model of Evolution (MEME) [32]. Remarkably, evidence of episodic diversifying selection was found at positions 149 and 427 (*p* < 0.1), suggesting that substitutions at these sites may be promoting an adaptative advantage to the Eurasia lineage. Interestingly, position 427 situated at the helicase conserved C-terminal domain appears as a polymorphic site in the alignment (A-V-E), with residue E highly conserved among most of the ASFVs with exception of the Eurasia lineage (A), supporting the potential evolutionary relevance of this site. Conversely, the change in the residue 149 appeared associated just to the isolate Estonia 2014. Furthermore, a deletion at residue 146 appears distinctive of all isolates belonging to the Eurasia lineage (Supplemental Figure S1). This finding contrasts with the multiple stop codons found at positions 169, 178, 202, 231, 300 and 352 disrupting multiple functional domains of the A859L protein in isolates RSA 2004, SPEC 57, Zaire, RSA W1 1999 and RSA 2 2008, suggesting that A859L may be no essential for the replication of ASFV, thus supporting the results of our study. Considering the high conservation of ASFV isolates at these sites, at this point, the most plausible explanations are that the stop codons may be mutations produced during the replication, a situation that may be expected considering the previously described low fidelity of the polymerase X of AFSV [33]. On the other hand, recombination may be another potential explanation. Interestingly, when we assessed the gene A859L by the algorithms GARD [34] and SBP [35], evidence of recombination was found supporting the existence of a potential break point at site 1155 of this gene, supporting the potential role of recombination in the evolution of A859L gene. 

### 3.2. Detection of A859L Transcription

**Figure 1:**
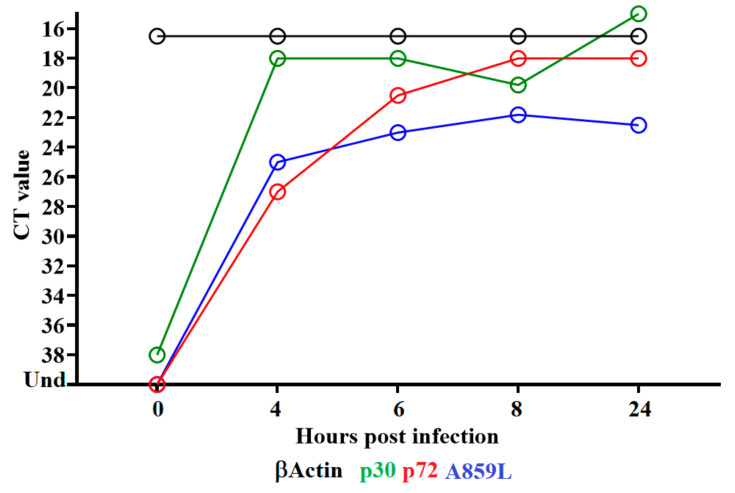
Expression profile of A859L gene of ASFV during in vitro infection of porcine macrophages. Reverse transcription followed by qPCR was used to evaluate the expression profile of the A859L gene during in vitro infection at different time points, up to 24 h. As a reference for this analysis, we use qPCRs to specifically detect the expression of genes encoding ASFV proteins p30 (early expression) and p72 (late expression). Additionally, the β-Actin gene was used as a control to evaluate the quality and levels of RNA during the infection at different time points.

To determine when the A859L gene is transcribed during the replication cycle, a time-course experiment was performed to analyze the kinetics of RNA transcription in primary swine macrophages infected with ASFV strain Georgia. Swine macrophage cultures were infected at an MOI = 1 with ASFV-G, and cell lysate samples were taken at 4, 6, 8 and 24 hpi. The presence of A859L RNA was detected by two-step RT-PCR, as described in the Material and Methods section. Transcription of A859L was detected at 4 hpi and remained stable until 24 hpi (Figure 1). The pattern of expression of the well-characterized ASFV early protein p30 (CP204L) and the late protein p72 (B646L) has been previously described and is used here as a reference of early and late transcription profiles, respectively. The expression of A859L is transiently detected throughout the timepoints suggesting that it is neither a late nor an early protein. This suggests that the ASFV A859L gene encodes for a protein that is expressed throughout the virus replication cycle.

### 3.3. Development of the ASFV-G-ΔA859L Deletion Mutant

The relative level of conservation of the A859L gene among different ASFV isolates and its predicted function as an RNA helicase [31] suggest that A859L may be involved in critical virus functions.

**Figure 2:**
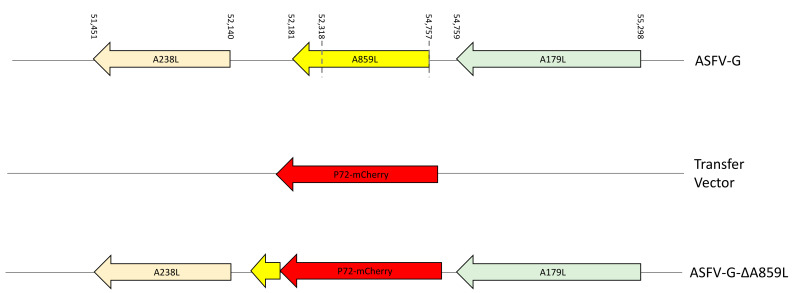
Schematic for the development of ASFV-G-∆A859L. The transfer vector contains the p72 promoter and a mCherry cassette; the gene positions are indicated. The homologous arms were designed to have flanking ends to both sides of the deletion/insertion cassette. The nucleotide positions of the area that was deleted in the ASFV-G genome are indicated by the dashed lines. The resulting ASFV-G-∆A859L virus with the cassette inserted is shown on the bottom.

To evaluate A859L during ASFV replication in cell cultures and in infected animals, a recombinant deletion mutant of the highly virulent ASFV Georgia 2007 isolate (ASFV-G) lacking the A859L gene was developed (ASFV-G-∆A859L). The A859L gene was deleted by swapping 813 amino acid residues in the A859L ORF with a p72mCherry cassette by homologous recombination [28]. A region spanning 2439-bp (between nucleotide positions 52,318 and 54,757) was deleted from the ASFV-G genome to delete the majority of the A859L gene by deleting the first 813 amino acids and leaving only the last 45 amino acids that are unlikely to be expressed due to the lack of a promoter and substituted with a 1226-bp cassette containing the p72mCherry construct (see Section 2.3) (Figure 2). The recombinant ASFV-G-∆A859L stock was purified after successive limiting dilution steps using primary swine macrophage cell cultures. The stock virus was produced by amplifying the virus obtained from the last purification round in primary swine macrophage cell cultures.

To evaluate the accuracy of the genetic modifications applied to the genome of ASFV-G-∆A859L as well as the integrity of the remaining virus genome, the full genome sequence was obtained by NGS using an Illumina NextSeq^®^ 500, a total of 3,433,439 reads aligned to the reference genome, and the comparative analysis of ASFV-G-∆A859L and ASFV-G genomes confirmed a deletion of 2439 nucleotides, in agreement with the designed genomic modifications. Additionally, the ASFV-G-∆A859L genome possesses an insertion of 1226 nucleotides consistent with the insertion of the p72-mCherry cassette sequence. No additional genomic differences were detected between ASFV-G-∆A859L and ASFV-G, confirming that no undesired changes were introduced during the process of production and purification of ASFV-G-∆A859L. In addition, NGS also indicated the absence of parental ASFV-G genome as a potential contaminant in the ASFV-G-∆A859L stock.

### 3.4. Replication of ASFV-G-∆A859L in Primary Swine Macrophages

**Figure 3:**
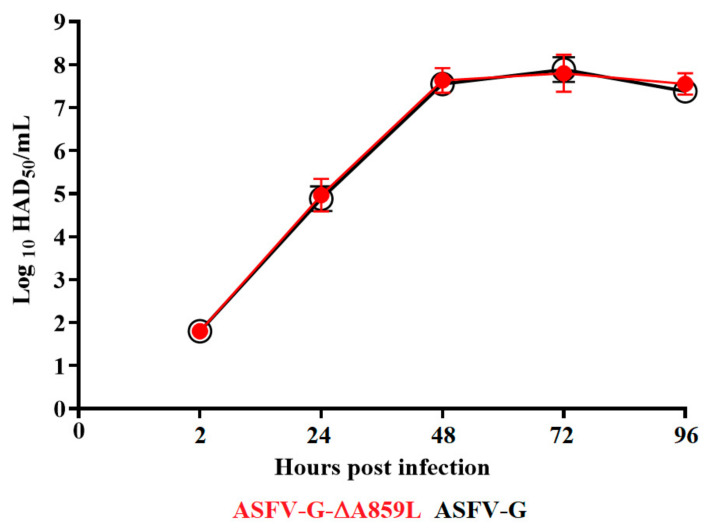
In vitro growth kinetics in primary swine macrophage cell cultures for ASFV-G-∆A859L and parental ASFV-G (MOI = 0.01). Samples were taken from three independent experiments at the indicated time points and titrated. Data represent the means and standard deviations of three replicas. Sensitivity using this methodology for detecting virus is ≥log10 1.8 HAD_50_/_mL_. No significant differences in viral yields between viruses were observed at any time point tested determined using the Holm–Sidak method (α = 0.05), without assuming a consistent standard deviation. All calculations were conducted using the software Graphpad Prism v8.

To understand the possible function of A859L during virus replication, the in vitro kinetics of ASFV-G-∆A859L replication was evaluated and compared to that of the parental ASFV-G. A multistep growth curve using swine macrophage cultures as a substrate was performed. Macrophages were infected (MOI of 0.01) with either recombinant ASFV-G-∆A859L or parental ASFV-G. Samples to evaluate virus yields were collected at 2, 24, 48, 72 and 96 h post-infection (pi). The results demonstrated that ASFV-G-∆A859L exhibited a very similar growth kinetic to that of the parental ASFV-G without any significant differences in virus yields at any of the evaluated times post-infection (Figure 3). 

Therefore, removal of the A859L gene from the ASFV-G genome does not affect the capability of ASFV-G-∆A859L to replicate in swine macrophages. This is an interesting result, considering the predicted function of the A859L gene as a helicase, particularly since other ASFV genes, also predicted to function as helicases, have been shown to be critical for virus replication [24].

### 3.5. Assessment of ASFV-G-∆A859L Virulence in Swine

**Figure 4:**
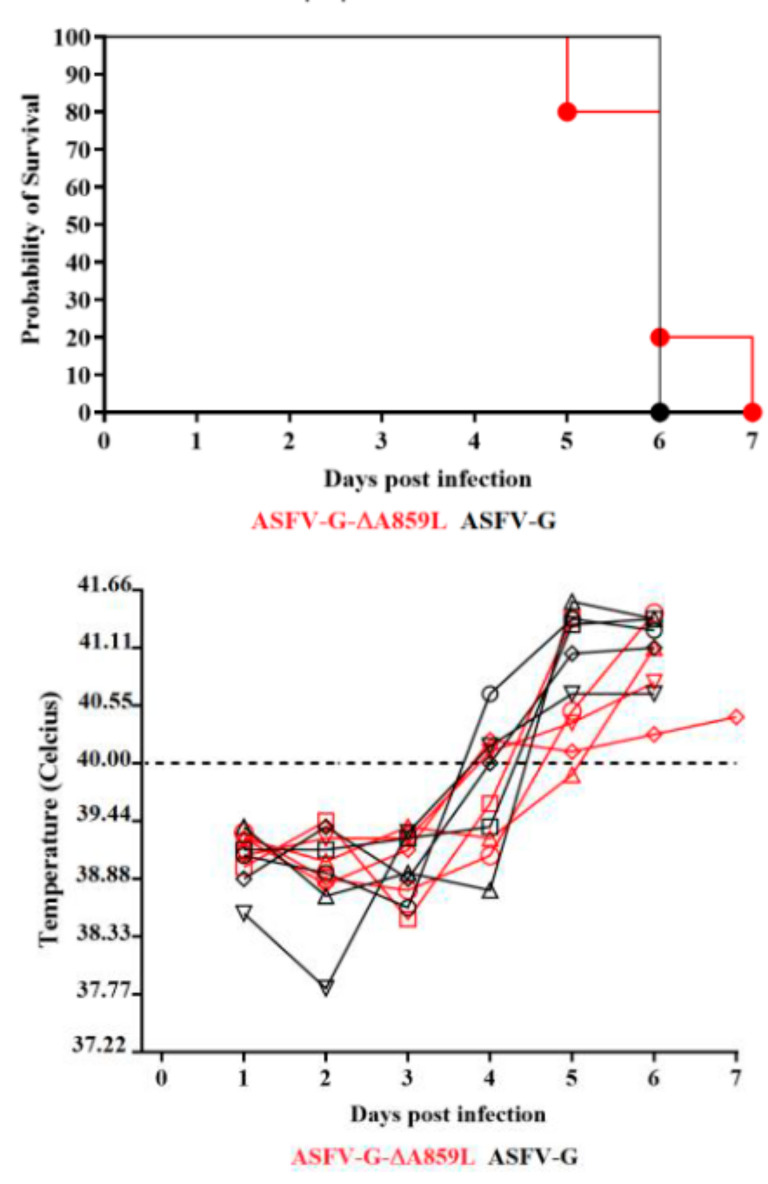
Evolution of mortality of animals (**top panel**) and body temperature, each symbol representing a different animal in the indicated group of animals (**bottom panel**) (5 animals/group) IM infected with 10^2^ HAD_50_ of either ASFV-G-∆A859L or parental ASFV-G. No significant differences were found in the survival course between groups of pigs using the Log-rank test (Mantel–Cox test). No statistical differences were found in body temperatures between pigs in both groups when evaluated by the Holm–Sidak method (α = 0.05). All calculations were conducted using the software GraphPad Prism version 8.

To assess the in vivo impact of removing the A859L gene from the ASFV-G genome, a group of five domestic pigs were IM inoculated with 10^2^ HAD_50_. A control group was also IM inoculated but with 10^2^ HAD_50_ of ASFV-G. As expected, all animals inoculated with virulent ASFV-G had a rise in body temperature (>40 °C) on 4–5 days pi. This was rapidly followed by the development of clinical disease (depression, anorexia, staggering gait, diarrhea and purple skin discoloration) (Table 1 and Figure 4). The clinical signs quickly evolved to a terminal disease, with all animals euthanized in extremis by day 7 pi.

All animals inoculated with ASFV-G-∆A859L developed a clinical disease indistinguishable from animals inoculated with ASFV-G. The timeline of the appearance of clinical signs and their severity were similar to those observed in the ASFV-G-inoculated animals. These results indicate that deletion of the A859L gene from the genome of ASFV-G does not affect virus virulence in domestic swine.

**Figure 5:**
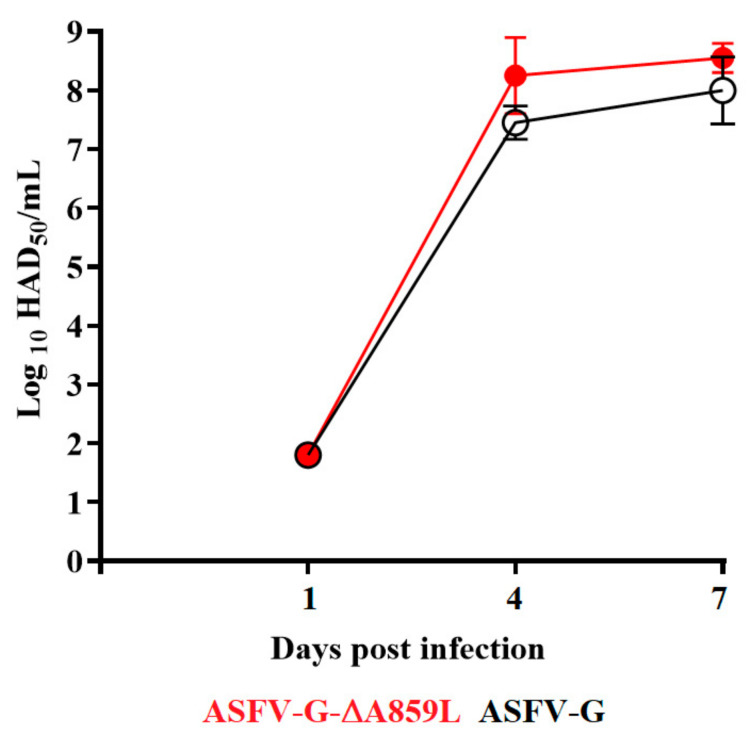
Viremia titers detected in pigs IM inoculated with 10^2^ HAD_50_ of either ASFV-G-∆A859L (filled symbols) or ASFV-G (empty symbols). Each symbol represents the average of animal titers in each of the groups. Sensitivity of virus detection: >log10 1.8 TCID_50_/_mL_. No significant differences in viremia values between both groups of pigs were found during the course of the experiment using the Holm–Sidak method (α = 0.05). All calculations were conducted on the software GraphPad Prism version 8.

Systemic virus replication in animals was assessed by determining viremia titers throughout the experimental period. Viremias in animals IM infected with parental ASFV-G had expected high titers (10^6.5^–10^7.5^ HAD_50_/_mL_) on day 4 pi, remaining high until day 7 pi, when all animals were euthanized. All animals inoculated with ASFV-G-∆A859L had viremia values similar to those inoculated with the parental virus by day 4 pi, reaching maximum titers by day 7 pi, when all animals were euthanized (Figure 5). Therefore, no statistical differences were found in the average of viremia titers at 4 dpi and undistinguishable between animals inoculated with either virus at 7dpi.

These results suggest that deletion of A859L from the genome of ASFV-G does not significantly affect virus replication or virulence in domestic swine. To further confirm that ASFV-G-∆ A859L was responsible for the virulent phenotype and viremia levels observed, the virus was isolated from the blood of ASFV-G-∆A859L-infected animals and analyzed by NGS. The results obtained by sequencing samples from three animals confirmed the absence of any significant differences with the full-length genome sequence of the ASFV-G-∆A859L stock. 

In summary, we determined that A859L is a non-essential gene since its deletion from the ASFV-G genome does not significantly alter virus replication either in vitro, in swine macrophage cultures, or during infection in vivo and, importantly, is not critical for ASFV virulence in swine. One potential explanation as to why A859L is non-essential would be the potential replacement of the helicase gene function by other ASFV genes with predicted helicase function [23,24]. The determination that A859L is non-essential increases the knowledge to determine which proteins could be deleted in the ASFV genome, which could allow the incorporation of a deletion of gene A859L into a minimal viral genome or incorporation with other deletions into next-generation ASFV vaccines. 

## Figures and Tables

**Table 1:** Swine survival and fever response following infection with ASFV-G-∆A859L and parental ASFV-G.

			Fever		
Virus (10^2^ HAD_50_)	No. of Survivors/Total	Mean Time to Death (±SD)	No. of Days to Onset (±SD)	Duration No. of Days (±SD)	Maximum Daily Temp, °C (±SD)
ASFV-G-∆A859L	0/5	6 (0.7)	4.8 (0.84)	1.4 (1.14)	40.9 (0.57)
ASFV-G	0/5	6 (0)	4.4 (0.54)	1.6 (0.55)	41.2 (0.35)

## Data Availability

Data is contained within the article

## Supplementary Materials

The following are available online at https://www.mdpi.com/article/10.3390/v14010010/s1, Figure S1: Amino acid diversity of protein encoded by A859L gene.
