# Supplementary material for: Evaluation of an ASFV RNA Helicase Gene A859L for Virus Replication and Swine Virulence
Source: Viruses. 2021 Dec 21;14(1):10. doi: 10.3390/v14010010 (PMC8777736; doi:10.3390/v14010010)
Supplement: Supplementary file 1 [file viruses-14-00010-s001.zip › viruses-1500788-supplementary.pdf]

Isolate	Genotype
Liv13/33	I
22653/Ca/2014	I
Georgia 2007/1	II
Estonia 2014	II
Tanzania/Rukwa/2017/1	II
HuB20	II
Warmbaths	III
SPEC 57	III
Warthog	IV
RSA W1 1999	IV
Ken05/Tk1	IX
R35	IX
Mkuzi 1979	VII
Uvira B53	VII
Malawi Lil-20/1	VIII
ASFV Ken.rie1	X
Kenya 1950	X
Ken06.Bus	X
Pretoriuskop/96/4	XX
RSA 2 2004	XX
Zaire	XX
RSA 2 2008	XXII

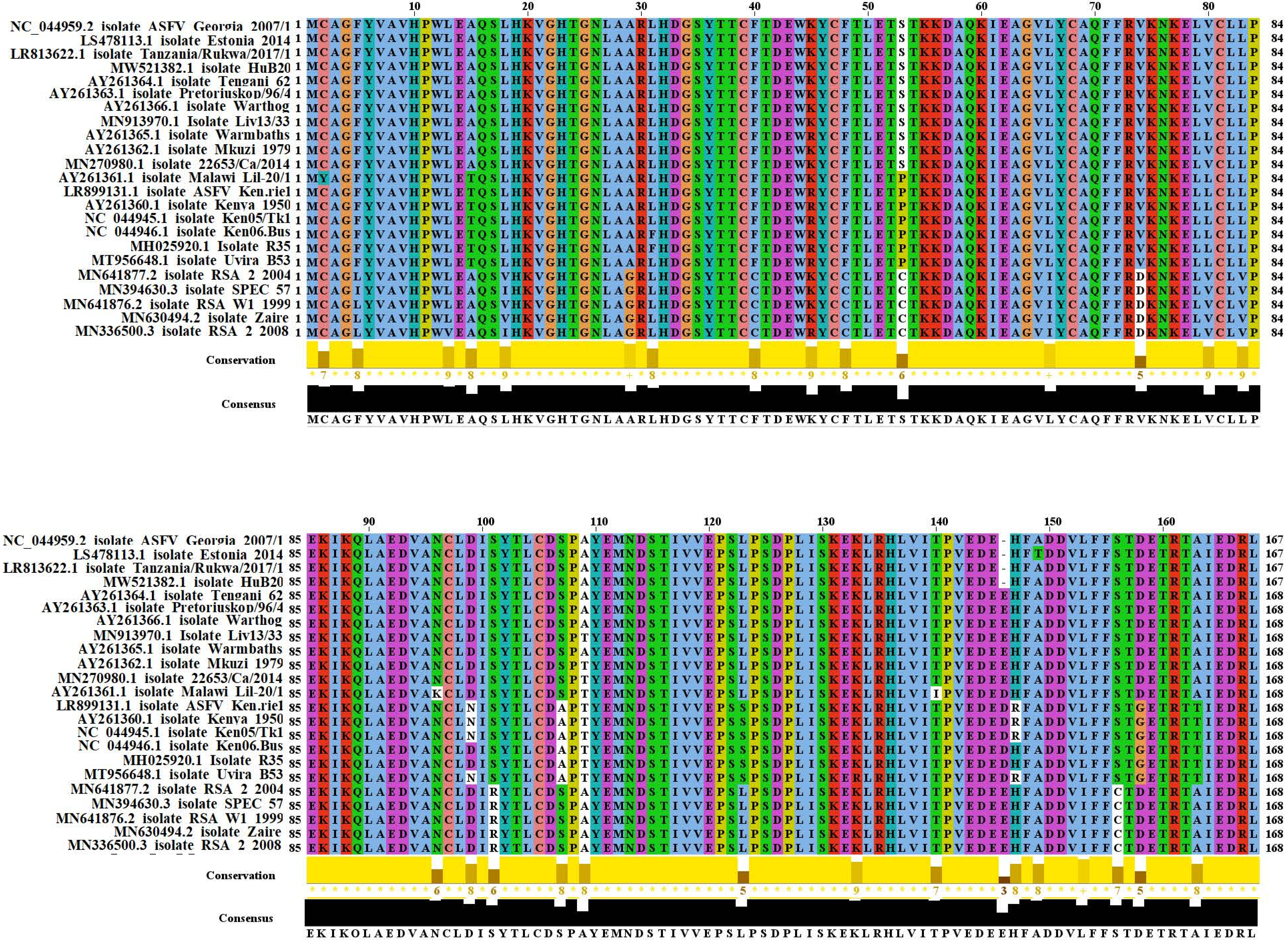

NC_044959.2 isolate ASFV Georgia 2007/1
LS478113.1 isolate Estonia 2014
LR813622.1 isolate Tanzania/Rukwa/2017/1
MW521382.1 isolate HuB20
AY261364.1 isolate Tengani 62
AY261363.1 isolate Pretoriuskop/96/4
AY261366.1 isolate Warthog
MN913970.1 Isolate Liv13/33
AY261365.1 isolate Warmbaths
AY261362.1 isolate Mkuzi 1979
MN270980.1 isolate 22653/Ca/2014
AY261361.1 isolate Malawi Lil-20/1
LR899131.1 isolate ASFV Ken.rie1
AY261360.1 isolate Kenya 1950
NC 044945.1 isolate Ken05/Tk1
NC 044946.1 isolate Ken06.Bus
MH025920.1 Isolate R35
MT956648.1 isolate Uvira B53
MN641877.2 isolate RSA 2 2004
MN394630.3 isolate SPEC 57
MN641876.2 isolate RSA W1 1999
MN630494.2 isolate Zaire
MN336500.3 isolate RSA 2 2008
10 20 30 40 50 60 70 80
Conservation
Consensus
MCAGFYVAVHPWLEAQSLHKVGHTGNLAARLHDGSYTTCFTDEWKYCFTLETSTKKDAQKIEAGVLYCAQFFRVKNKELVCLLP
90 100 110 120 130 140 150 160
Conservation
Consensus
EKIKQLAEDVANCLDISYTLCDSPAYEMNDSTIVVEPSLPSDPLISKEKLRHLVITPVEDEEHFADDVLFFSTDETRTAIEDRL

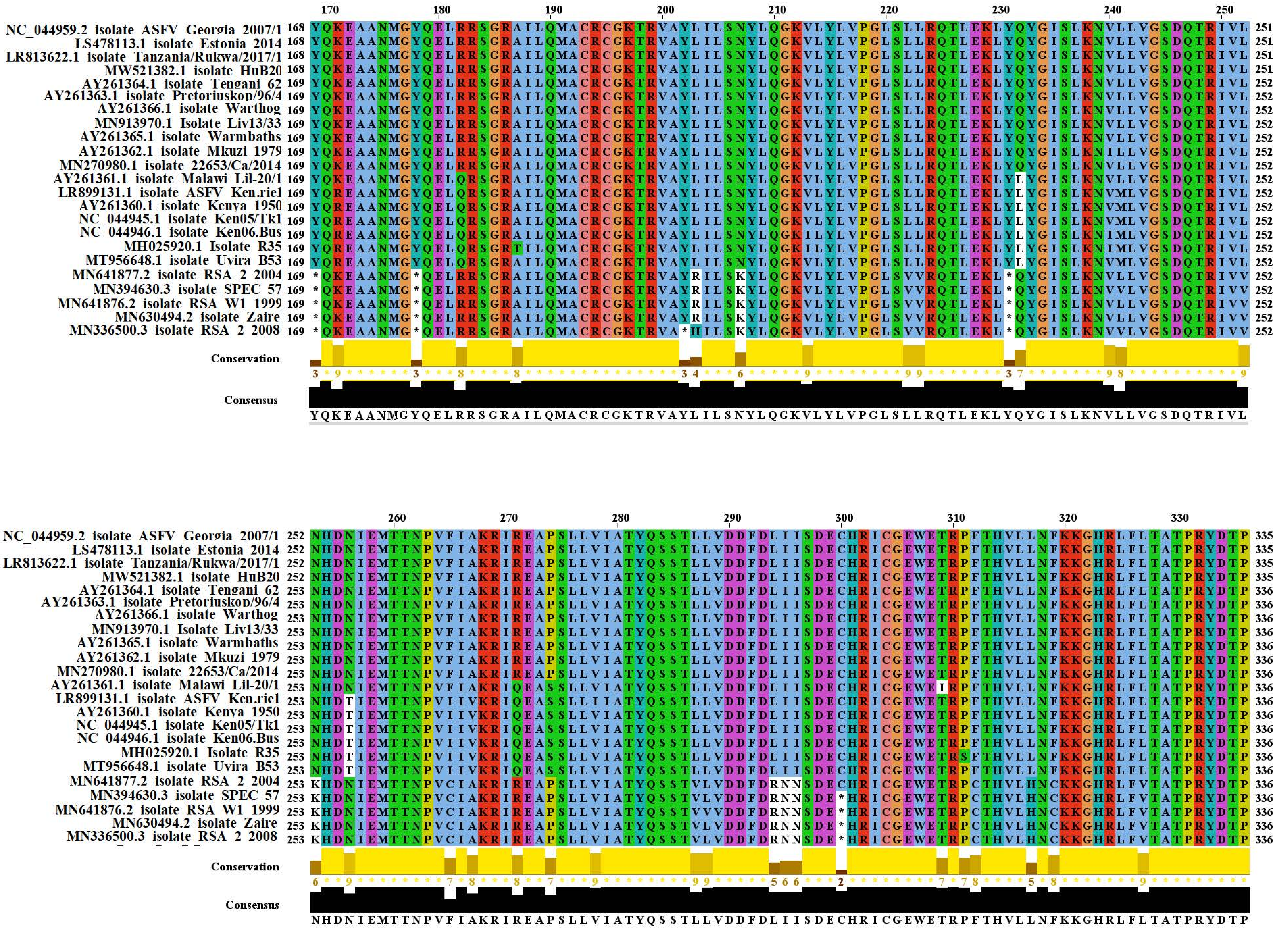

NC_044959.2 isolate ASFV Georgia 2007/1
LS478113.1 isolate Estonia 2014
LR813622.1 isolate Tanzania/Rukwa/2017/1
MW521382.1 isolate HuB20
AY261364.1 isolate Tengani 62
AY261363.1 isolate Pretoriuskop/96/4
AY261366.1 isolate Warthog
MN913970.1 Isolate Liv13/33
AY261365.1 isolate Warmbaths
AY261362.1 isolate Mkuzi 1979
MN270980.1 isolate 22653/Ca/2014
AY261361.1 isolate Malawi Lil-20/1
LR899131.1 isolate ASFV Ken.rie1
AY261360.1 isolate Kenya 1950
NC 044945.1 isolate Ken05/Tk1
NC 044946.1 isolate Ken06.Bus
MH025920.1 Isolate R35
MT956648.1 isolate Uvira B53
MN641877.2 isolate RSA 2 2004
MN394630.3 isolate SPEC 57
MN641876.2 isolate RSA W1 1999
MN630494.2 isolate Zaire
MN336500.3 isolate RSA 2 2008
Conservation
Consensus
YQKEAANMGYQELRRSGRAILQMACRCGKTRVAYLILSNYLQGKVLYLVPGLSLLRQTLEKLYQYGISLKNVLLVGSDQTRIVL
NHDNIEMTTNPVFIAKRIREAPSLLVIATYQSSTLLVDDFDLIISDECHRICGEWETRPFTHVLLNFKKGHRLFLTATPRYDTP

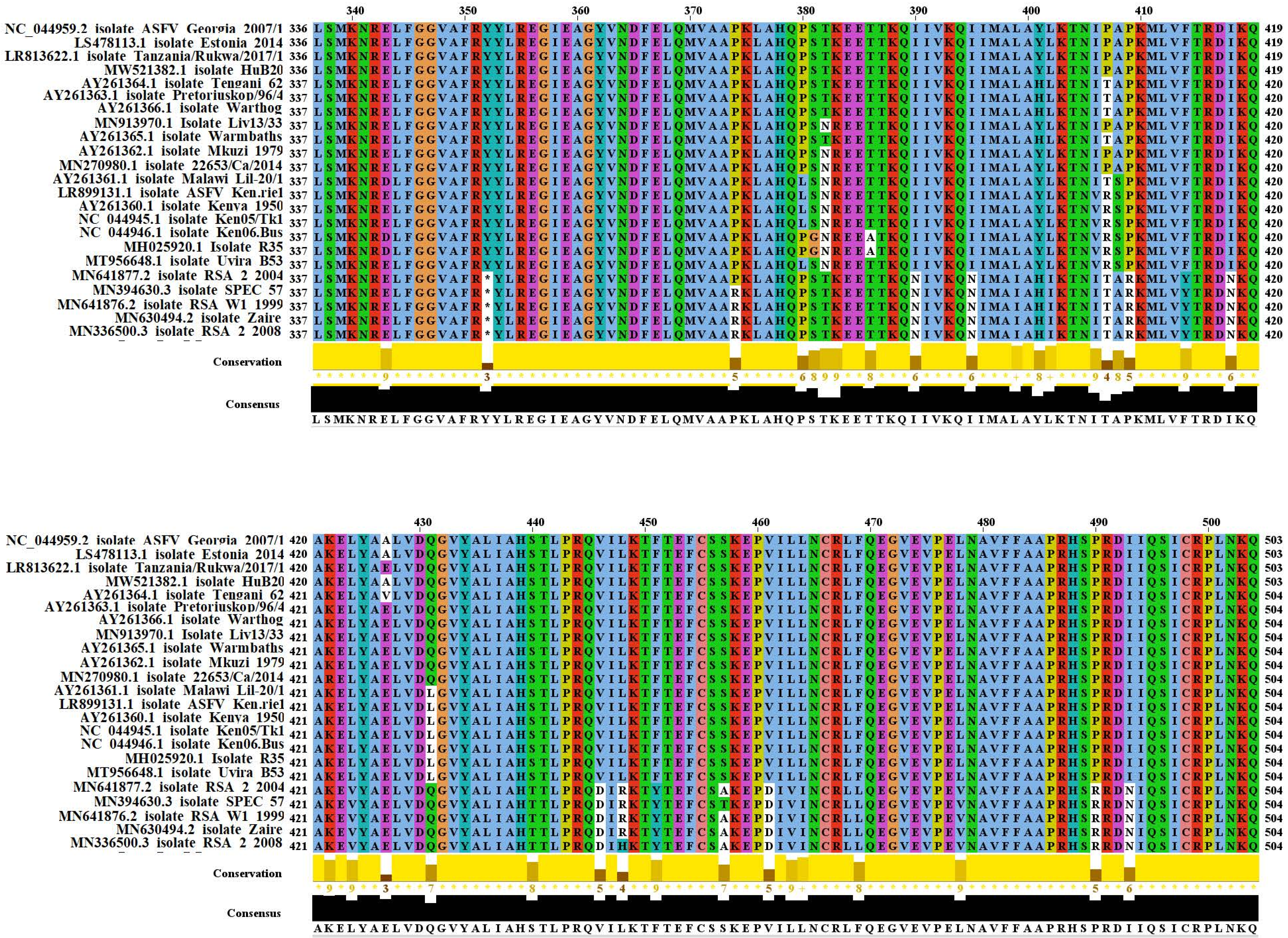
NC_044959.2 isolate ASFV Georgia 2007/1
LS478113.1 isolate Estonia 2014
LR813622.1 isolate Tanzania/Rukwa/2017/1
MW521382.1 isolate HuB20
AY261364.1 isolate Tengani 62
AY261363.1 isolate Pretoriuskop/96/4
AY261366.1 isolate Warthog
MN913970.1 Isolate Liv13/33
AY261365.1 isolate Warmbaths
AY261362.1 isolate Mkuzi 1979
MN270980.1 isolate 22653/Ca/2014
AY261361.1 isolate Malawi Lil-20/1
LR899131.1 isolate ASFV Ken.rie1
AY261360.1 isolate Kenya 1950
NC_044945.1 isolate Ken05/Tk1
NC_044946.1 isolate Ken06.Bus
MH025920.1 Isolate R35
MT956648.1 isolate Uvira B53
MN641877.2 isolate RSA_2_2004
MN394630.3 isolate SPEC_57
MN641876.2 isolate RSA_W1_1999
MN630494.2 isolate Zaire
MN336500.3 isolate RSA_2_2008
Conservation
Consensus
LSMKNRELFGGVAFRYYLREGIEAGYVNDFELQMVAAPKLAHQPSTKEETTKQIIVKQIIMALAYLKTNITAPKMLVFTRDIKQ
AKELYAELVDQGVYALIAHSTLPRQVILKTFTEFCSSKEPVILLNCRLFQEGVEVPELNAVFFAAPRHSPRDIIQSICRPLNKQ

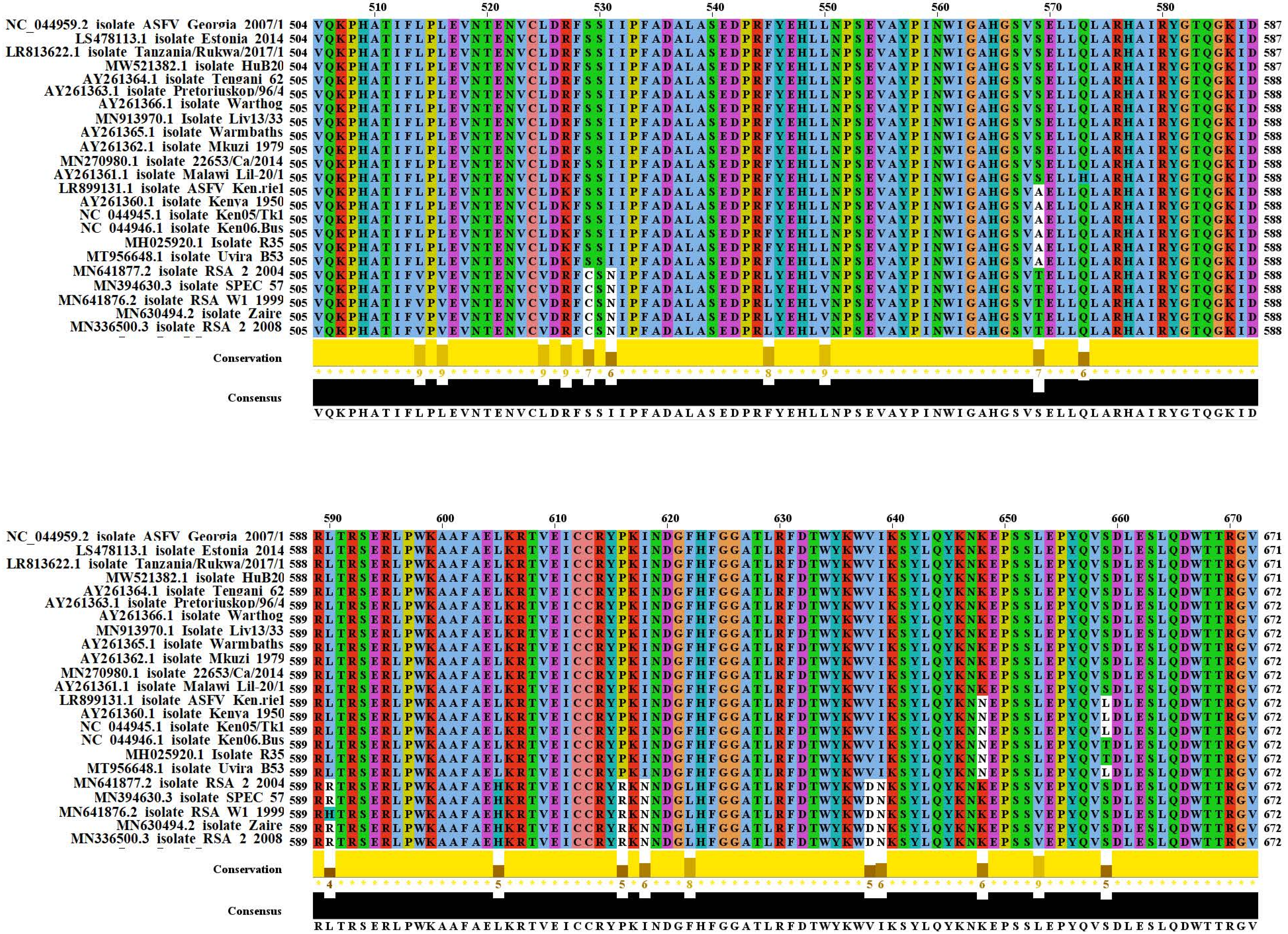
NC_044959.2 isolate ASFV Georgia 2007/1
LS478113.1 isolate Estonia 2014
LR813622.1 isolate Tanzania/Rukwa/2017/1
MW521382.1 isolate HuB20
AY261364.1 isolate Tengani 62
AY261363.1 isolate Pretoriuskop/96/4
AY261366.1 isolate Warthog
MN913970.1 Isolate Liv13/33
AY261365.1 isolate Warmbaths
AY261362.1 isolate Mkuzi 1979
MN270980.1 isolate 22653/Ca/2014
AY261361.1 isolate Malawi Lil-20/1
LR899131.1 isolate ASFV Ken.rie1
AY261360.1 isolate Kenya 1950
NC 044945.1 isolate Ken05/Tk1
NC 044946.1 isolate Ken06.Bus
MH025920.1 Isolate R35
MT956648.1 isolate Uvira B53
MN641877.2 isolate RSA 2 2004
MN394630.3 isolate SPEC 57
MN641876.2 isolate RSA W1 1999
MN630494.2 isolate Zaire
MN336500.3 isolate RSA 2 2008
Conservation
Consensus
VQKPHATIFLPLEVNTENVCLDRFSSIIPFADALASEDPRFYEHLLNPSEVAYPINWIGAHGSVSELLQLARHAIRYGTQGKID
RLTRSERLPWKAAFAELKRTVEICCRYPKINDGFHFGGATLRFDTWYKWVIKSYLQYKNKEPSSLEPYQVSDLESLQDWTTRGV

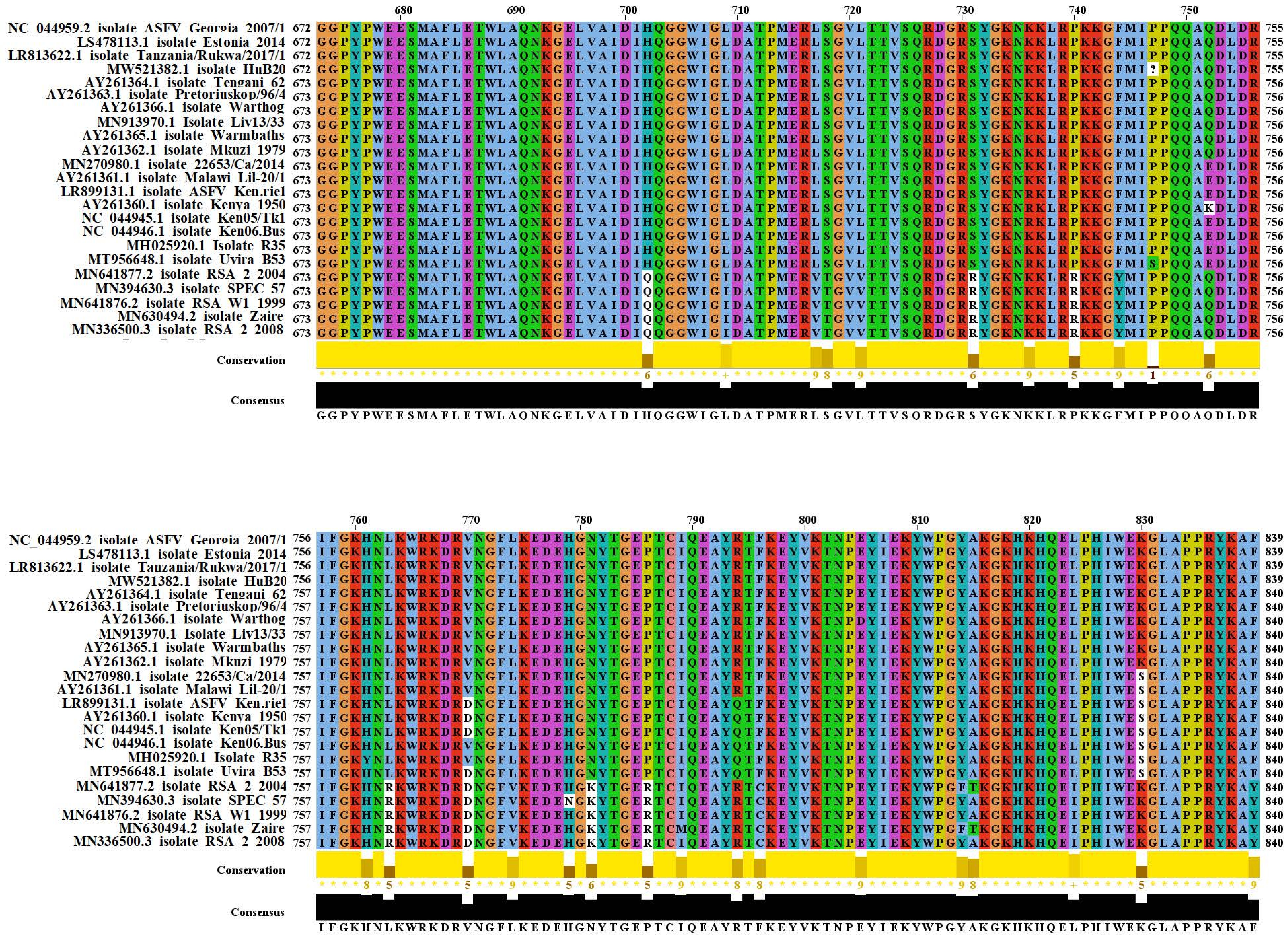

NC_044959.2 isolate ASFV Georgia 2007/1
LS478113.1 isolate Estonia 2014
LR813622.1 isolate Tanzania/Rukwa/2017/1
MW521382.1 isolate HuB20
AY261364.1 isolate Tengani 62
AY261363.1 isolate Pretoriuskop/96/4
AY261366.1 isolate Warthog
MN913970.1 Isolate Liv13/33
AY261365.1 isolate Warmbaths
AY261362.1 isolate Mkuzi 1979
MN270980.1 isolate 22653/Ca/2014
AY261361.1 isolate Malawi Lil-20/1
LR899131.1 isolate ASFV Ken.rie1
AY261360.1 isolate Kenya 1950
NC 044945.1 isolate Ken05/Tk1
NC 044946.1 isolate Ken06.Bus
MH025920.1 Isolate R35
MT956648.1 isolate Uvira B53
MN641877.2 isolate RSA 2 2004
MN394630.3 isolate SPEC 57
MN641876.2 isolate RSA W1 1999
MN630494.2 isolate Zaire
MN336500.3 isolate RSA 2 2008
Conservation
Consensus
GGPYPWEESMAFLETWLAQNKGELVAIDIHQGGWIGLDATPMERLSGVLTTVSQRDGRSYGKNKKLRPKKGFMIPPQQAQDLDR
IFGKHNLKWRKDRVNGFLKEDEHGNYTGEPTCIQEAYRTFKEYVKTNPEYIEKYWPGYAKGKHKHQELPHIWEKGLAPPRYKAF

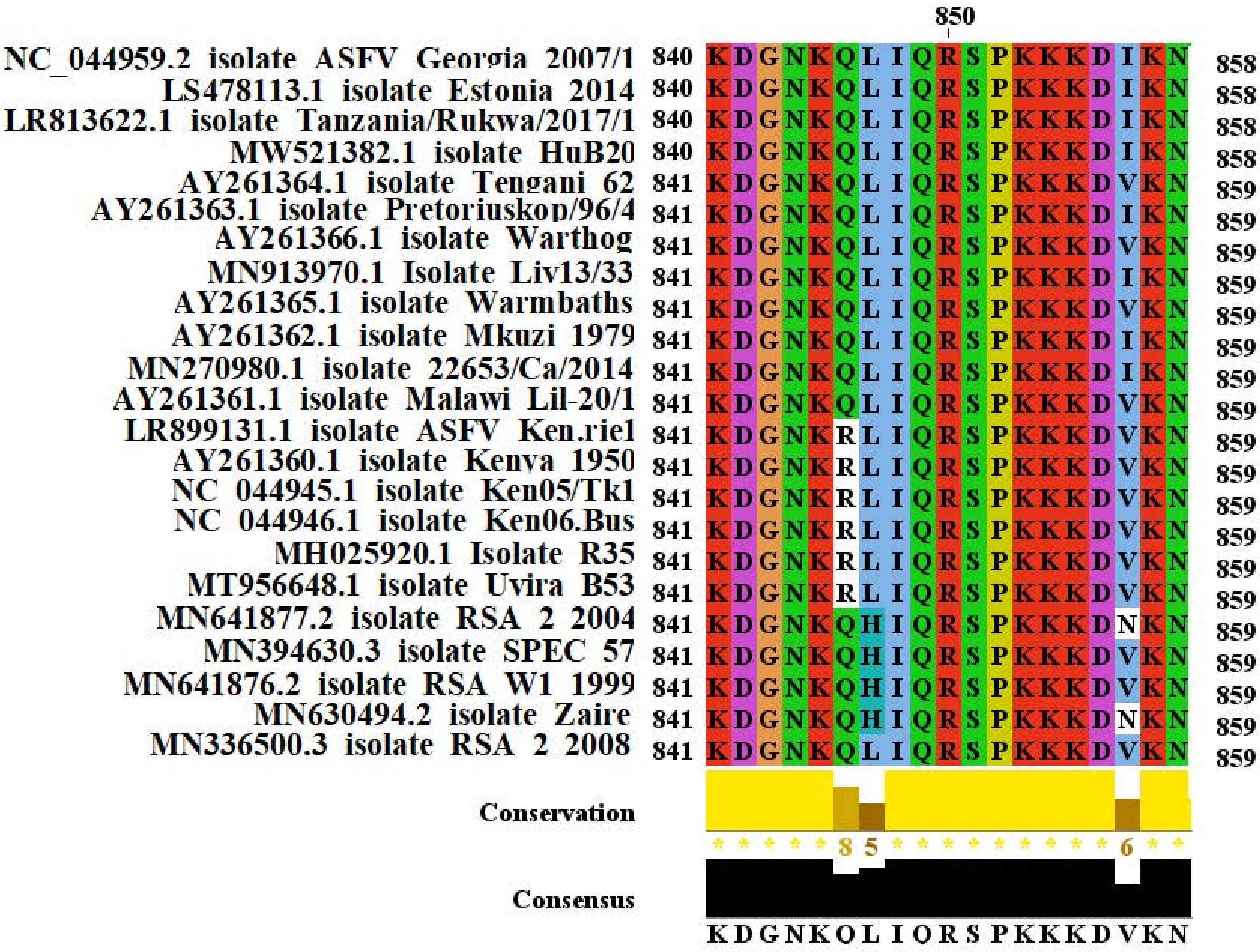

850
NC_044959.2 isolate ASFV Georgia 2007/1 840 KDGNKQLIQRSPKKKDIKN 858
LS478113.1 isolate Estonia 2014 840 KDGNKQLIQRSPKKKDIKN 858
LR813622.1 isolate Tanzania/Rukwa/2017/1 840 KDGNKQLIQRSPKKKDIKN 858
MW521382.1 isolate HuB20 840 KDGNKQLIQRSPKKKDIKN 858
AY261364.1 isolate Tengani 62 841 KDGNKQLIQRSPKKKDVKN 859
AY261363.1 isolate Pretoriuskop/96/4 841 KDGNKQLIQRSPKKKDIKN 859
AY261366.1 isolate Warthog 841 KDGNKQLIQRSPKKKDVKN 859
MN913970.1 Isolate Liv13/33 841 KDGNKQLIQRSPKKKDIKN 859
AY261365.1 isolate Warmbaths 841 KDGNKQLIQRSPKKKDVKN 859
AY261362.1 isolate Mkuzi 1979 841 KDGNKQLIQRSPKKKDIKN 859
MN270980.1 isolate 22653/Ca/2014 841 KDGNKQLIQRSPKKKDIKN 859
AY261361.1 isolate Malawi Lil-20/1 841 KDGNKQLIQRSPKKKDVKN 859
LR899131.1 isolate ASFV Ken.rie1 841 KDGNKRLIQRSPKKKDVKN 859
AY261360.1 isolate Kenya 1950 841 KDGNKRLIQRSPKKKDVKN 859
NC 044945.1 isolate Ken05/Tk1 841 KDGNKRLIQRSPKKKDVKN 859
NC 044946.1 isolate Ken06.Bus 841 KDGNKRLIQRSPKKKDVKN 859
MH025920.1 Isolate R35 841 KDGNKRLIQRSPKKKDVKN 859
MT956648.1 isolate Uvira B53 841 KDGNKRLIQRSPKKKDVKN 859
MN641877.2 isolate RSA 2 2004 841 KDGNKQHIQRSPKKKDNKN 859
MN394630.3 isolate SPEC 57 841 KDGNKQHIQRSPKKKDVKN 859
MN641876.2 isolate RSA W1 1999 841 KDGNKQHIQRSPKKKDVKN 859
MN630494.2 isolate Zaire 841 KDGNKQHIQRSPKKKDNKN 859
MN336500.3 isolate RSA 2 2008 841 KDGNKQLIQRSPKKKDVKN 859
Conservation
* * * * * 8 5 * * * * * * * * * 6 * *
Consensus
KDGNKQLIQRSPKKKDVKN

Supplemental Figure S1. Amino acid diversity of protein encoded by A859L gene. Twenty-three protein sequences representing the genetic diversity of gene A859L of ASFV at GenBank database were used to conduct this alignment. To evaluate the nature of the replacements at numerous residues, conservation scores based on the biological properties of each amino acid were included, being the lower scores associated with more divergent replacements. Symbols (*) indicate residue conservation or (+) replacement for an amino acid with similar properties. Analysis was conducted on the Jalview software version 2.11.1.3, using the Clustal W algorithm.
